# NUCLIZE for quantifying epigenome: generating histone modification data at single-nucleosome resolution using genuine nucleosome positions

**DOI:** 10.1186/s12864-019-5932-6

**Published:** 2019-07-02

**Authors:** Daoshan Zheng, Justyna Trynda, Zhifu Sun, Zhaoyu Li

**Affiliations:** 1 Department of Cancer Biology and Mayo Clinic Comprehensive Cancer Center, Mayo Clinic, 4500 San Pablo Road, Griffin 210, Jacksonville, FL 32224 USA; 2 Bioinformatics Core and Mayo Clinic Comprehensive Cancer Center, Mayo Clinic, 4500 San Pablo Road, Griffin 210, Jacksonville, FL 32224 USA

**Keywords:** NUCLIZE, Histone modification, Nucleosome positioning, Quantitative epigenomics, Graph epigenome

## Abstract

**Background:**

Defining histone modification at single-nucleosome resolution provides accurate epigenomic information in individual nucleosomes. However, most of histone modification data deposited in current databases, such as ENCODE and Roadmap, have low resolution with peaks of several kilo-base pairs (kb), which due to the technical defects of regular ChIP-Seq technology.

**Results:**

To generate histone modification data at single-nucleosome resolution, we developed a novel approach, NUCLIZE, using synergistic analyses of histone modification data from ChIP-Seq and high-resolution nucleosome mapping data from native MNase-Seq. With this approach, we generated quantitative epigenomics data of single and multivalent histone modification marks in each nucleosome. We found that the dominant trivalent histone mark (H3K4me3/H3K9ac/H3K27ac) and others showed defined and specific patterns near each TSS, indicating potential epigenetic codes regulating gene transcription.

**Conclusions:**

Single-nucleosome histone modification data render epigenomic data become quantitative, which is essential for investigating dynamic changes of epigenetic regulation in the biological process or for functional epigenomics studies. Thus, NUCLIZE turns current epigenomic mapping studies into genuine functional epigenomics studies with quantitative epigenomic data.

**Electronic supplementary material:**

The online version of this article (10.1186/s12864-019-5932-6) contains supplementary material, which is available to authorized users.

## Background

Chromatin immunoprecipitation coupled high-throughput sequencing (ChIP-Seq) has greatly improved the genome-wide understanding of chromatin structures, particularly, histone modification in the biological systems. The nucleosome is the fundamental unit of chromatin architecture. Histone modification resides in individual nucleosomes. Thus, high-resolution histone modification data should display all histone modification information in each nucleosome in the epigenome. However, the key defect of current ChIP-Seq technology for the histone modification analysis is the low resolution. ChIP-Seq cannot identify histone modification at single-nucleosome resolution, even when micrococcal nuclease (MNase) is used to digest crosslinked chromatin. This is due to the major technical defect of the ChIP-Seq technology using formaldehyde to crosslink chromatin, which introduces broad (greater than a nucleosome) or non-specific signals of histone modification, regardless of sonication or MNase digestion; thus, ChIP-Seq generates broad peaks of histone modification ranging from a few hundred to thousand base pairs (bp) for a single histone mark (Additional file 1: Figure S1), which could not be resolved by peak-calling algorithms, such as MACS, HOMER, and SICER [1–3]. This low-resolution histone modification data make it difficult to conduct quantitative and functional studies of histone modification signals, especially when comparing multivalent and/or different sizes of histone modification peaks at the same genomic loci between two datasets.

### Implementation

**Fig. 1:**
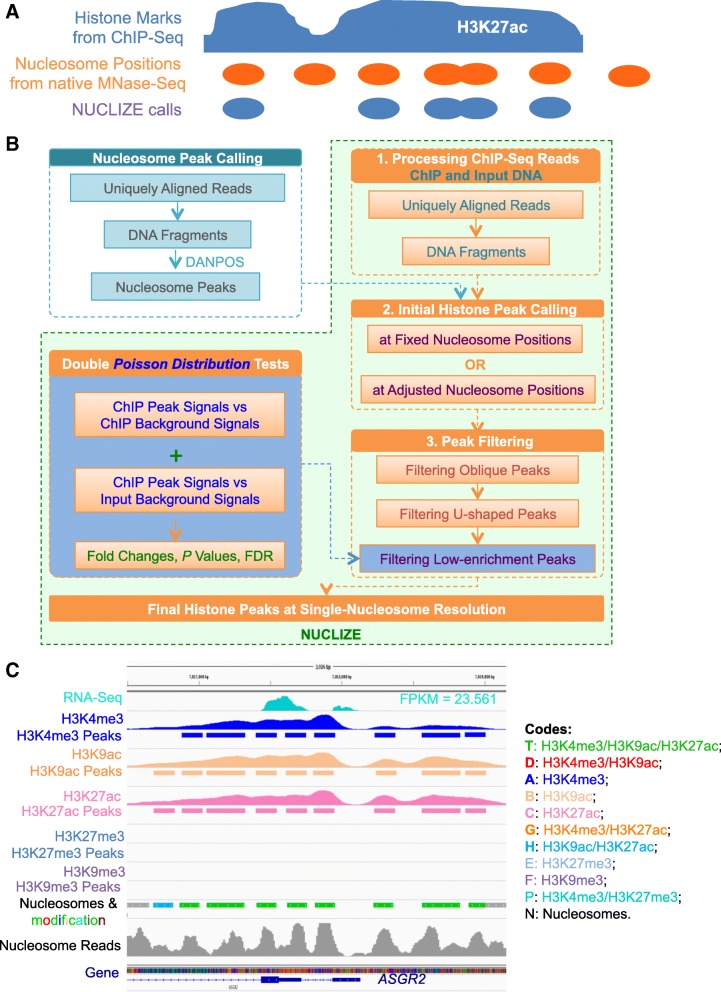
NUCLIZE: defining histone modification at single-nucleosome resolution. (**a**) Schematic view of the NUCLIZE approach for re-annotating histone modification data at single-nucleosome resolution. (**b**) The NUCLIZE Workflow. (**c**) Examples of single and multivalent histone modification signals at each nucleosome position near the TSS of a highly expressed gene, *ASGR2*, in HepG2 cells. Each color represents one single or multivalent histone modification mark in each nucleosome from combined analysis of all five histone marks

Nucleosome positioning has been under-evaluated in the analysis of histone modification data from ChIP-Seq. Thus, we developed a novel approach, NUCLIZE, to define histone modification at single-nucleosome resolution by synergistic analyses of histone modification data from ChIP-Seq and high-resolution nucleosome mapping data from native MNase-Seq (Fig. 1a). Our NUCLIZE approach includes three major components, regular ChIP-Seq data of histone modification, native MNase-Seq data of nucleosome positions, and our novel algorithm, NUCLIZE, for calling single-nucleosome histone modification peaks from ChIP-Seq reads based on genuine nucleosome positions from our nucleosome mapping data without any crosslinking in the same cells as those for ChIP-Seq (Fig. 1a). Our nucleosome maps were generated from the digestion of isolated native chromatin with MNase with the approach described previously [4, 5]. We used the human liver cancer cell line, HepG2, as our model for the NUCLIZE analysis. From native MNase-Seq, we generated about 300 million uniquely aligned pair-end reads of 101 bp each to completely cover each nucleosome of 147 bp for at least ten times in the human genome. From ChIP-Seq, we generated over 30 million uniquely aligned pair-end reads of 50 bp for H3K4me3, H3K9ac, H3K9me3, H3K27ac, and H3K27me3. Figure 1b shows the NUCLIZE workflow. First, we performed the nucleosome peak calling using DANPOS [6] with default parameters to obtain native nucleosome positions. Next, we re-analyzed histone modification reads from ChIP-Seq using NUCLIZE. Briefly, the main process of NUCLIZE included three steps: 1) Re-processing histone modification reads from ChIP-Seq for both ChIP and input DNA to generate DNA fragments. 2) Initial histone peak calling: calculating the enrichment of ChIP reads at each nucleosome position based on fixed nucleosome positions from DANPOS calls and calling initial histone peaks with at least four more reads. 3) Peak filtering: three filtering procedures were applied to filter out the noise or anomalous peaks introduced by crosslinking, including oblique and U-shaped peaks, based on read enrichment and distributions or peak shapes within each nucleosome position, and filtering out low-enrichment peaks using double Poisson distribution tests between both peak signals and local background signals from ChIP reads and between peak signals from ChIP reads and background signals from input reads; peaks were filtered by *P* value ≤0.01 and fold changes ≥4-fold from ChIP to input signals in both tests. Additionally, the Benjamini-Hochberg method [7] was applied to calculate the false discovery rate (FDR) for local background signals from ChIP reads and input reads for each peak, and FDR ≤ 0.01 were used to filter peaks. Detailed computational calculation formulations and procedures can be found in the Supplemental Experimental Procedures. Multivalent histone marks in the same nucleosome were calculated based on the nucleosome location for each histone mark. Other details of the methods are available in the Additional file.

With these single-nucleosome high-resolution histone modification data, we will be able to generate quantitative epigenomic data of multivalent histone modification marks at single-nucleosome resolution, which is a critical step towards genuine functional epigenomics studies.

## Results

### Single-nucleosome histone modification data in human cells

With our approach, we obtained single-nucleosome resolution of histone modification data, including H3K4me3, H3K9ac, H3K9me3, H3K27ac, and H3K27me3, in HepG2 cells. Figure 1c and Additional file 1: Figure S2A show multivalent histone modification signals at each nucleosome position near the transcription start site (TSS) of a highly expressed gene (*ASGR2*), a weakly expressed gene (*RTTN*), and a silent gene (*LINC01182*) in HepG2 cells. Each color represents a nucleosome with single or multivalent histone modification. To validate our NUCLIZE approach, we performed quantitative PCR to measure the enrichment of histone modification in both NUCLIZE-called and -discarded peaks for over 200 randomly selected sites for each mark and observed 100% consistency with the NUCLIZE analysis (Additional file 1: Figure S2B). These data indicate that defined and/or differential numbers and patterns of histone marks in a string of nucleosomes near TSS may delicately modulate gene expression.

### Intra- and cross-platform analysis using NUCLIZE

Compared to large amount of histone modification mapping data that have been generated in the public databases, high-resolution nucleosome mapping has only been completed in a few human normal and cancer cell types, including CD4+ and CD8+ T-cells [8, 9], CD34+ hematopoietic stem cells and their derivatives, CD36+ erythroid lineage cells [10], IMR90 cells [11], human sperm [12, 13], human lymphoblastoid cell lines [14, 15], MCF7 [16], HEK293 [17], and K562 [15]; however, the qualities of these nucleosome mapping data varied and some data were from MNase digestion of crosslinked chromatin. Thus, to obtain accurate nucleosome mapping data, native MNase-Seq need to be performed in those cells with ChIP-Seq histone modification data that are deposited in the public databases, so that we will be able to re-annotate these data into single-nucleosome resolution using our NUCLIZE approach. Therefore, the major concern is the cross-platform issue for analyzing ChIP-Seq data and MNase-Seq data from different groups; in other words, a major issue occurs in this case regarding whether the data generated in same cells from different groups (cross-platform data) are similar to those data generated in the same group (intra-platform), such as our data. To address this, we performed the NUCLIZE analysis for two sets of histone modification data in HepG2 cells from two different groups in the ENCODE data using our nucleosome mapping data and compared all nucleosomes with histone modification signals between the ENCODE data and our own data as shown in Fig. 1c. The principal component analysis (PCA) showed that our data of single and multivalent histone marks in each nucleosome were highly similar with both sets of ENCODE data compared to non-correlated data from MCF7 cells (Additional file 1: Figure S3). These data indicate that our NUCLIZE approach to define histone modification data into single-nucleosome resolution is promising for the cross-platform analysis.

### Quantitative signals of single and multivalent histone modification in each nucleosome

**Fig. 2:**
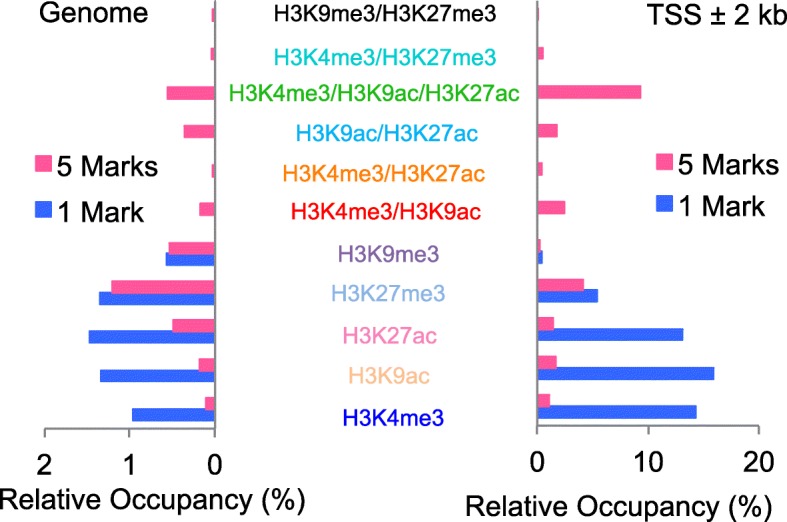
Distributions of single-nucleosome histone modification signals in the genome Relative occupancy of histone modification signals in each nucleosome with individual marks (1 Mark) and with combined marks (5 Marks) of five histone marks together in the whole genome and within ±2 kb of the TSS of genes in HepG2 cells

We obtained about 15 million nucleosome positions in HepG2 cells and about 3.7% of nucleosomes (571,511) had histone modification. When analyzing individual histone marks in each nucleosome, we found that each histone mark (only one mark at a time, 1 Mark in Fig. 2) covered about 0.5–1.5% of total nucleosomes in the whole genome, but there was up to 16% enrichment within ±2 kb of the TSS; and near the TSS, only H3K4me3, H3K9ac, and H3K27ac were highly enriched, H3K27me3 was weakly located, and H3K9me3 was barely found at ±2 kb of the TSS. With combinational analysis of all five histone marks in each nucleosome (5 Marks in Fig. 2), we found 11 single and multivalent histone marks with detectable signals in each nucleosome throughout the whole genome, including a trivalent mark (H3K4me3/H3K9ac/H3K27ac, “T”), five bivalent marks (H3K4me3/H3K9ac, H3K4me3/H3K27ac, H3K9ac/H3K27ac, and H3K4me3/H3K27me3, H3K9me3/H3K27me3), and five single marks (H3K4me3, H3K9ac, H3K9me3, H3K27ac, and H3K27me3), ranging from 0.02 to 1.2% of multivalent histone marks in the genome; most of them also appeared within ±2 k base pairs (kb) of the TSS, but only the trivalent mark “T” was mostly enriched within ±2 kb of the TSS. Compared to the data from the calculation of individual histone marks (1 Mark in Fig. 2), about 70–80% H3K4me3, H3K9ac, and H3K27ac within ±2 kb of the TSS were regulated coordinately as multivalent histone marks in each nucleosome, in which the trivalent mark “T” was highly enriched, suggesting this multivalent histone modification mark “T” may play a key role in the regulation of gene transcription. Interestingly, as suppression marks, H3K9me3 and H3K27me3 signals were barely co-regulated together near TSS (Fig. 2) and most of these marks located within ±10–1000 kb outside the TSS (Additional file 1: Figure S4).

### Epigenetic codes? Patterns of a string of single-nucleosome histone modification signals near TSS

**Fig. 3:**
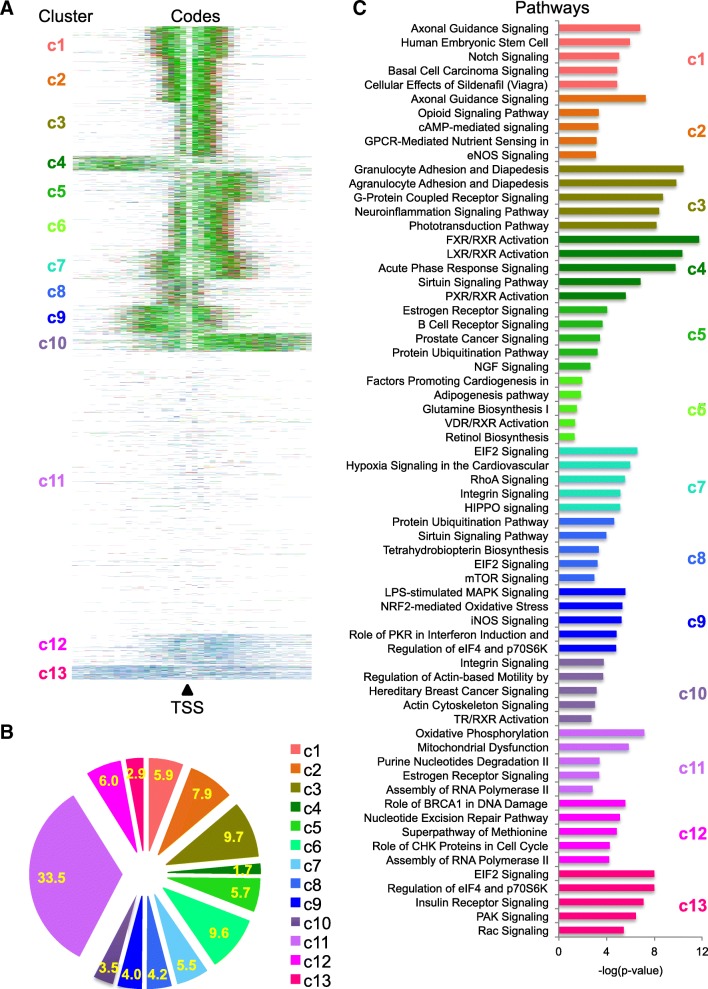
Patterns of a string of single-nucleosome histone modification signals near TSS. (**a**) Patterns of histone modification codes near TSS by the K-means clustering. Each color represents one single or multivalent histone modification mark in each nucleosome from combined analysis of all five histone marks shown in Fig. 1c. (**b**) Percentages of genes with and without histone marks from the clustering analysis in (**a**). (**c**) The top 5 canonical pathways could be regulated by genes in each cluster using Ingenuity

Whether there are epigenetic codes for gene regulation has been argued for a while, and related research has mostly focused on a single histone protein or a single-nucleosome locus [18–20]. Our high-resolution histone modification data at single-nucleosome resolution provided us a great opportunity to tackle this question by expanding it to a consecutive set of quantitative histone marks in a string of nucleosomes at gene regulatory regions. To determine whether these defined numbers or lengths of single and multivalent histone marks have patterns or whether there are epigenetic codes for regulating gene expression at the genomic regulatory regions surrounding the TSS, we evaluated the histone modification codes for each gene in HepG2 cells. Here, a code is defined as a string of nucleosome positions with histone marks surrounding the TSS of each gene. We found that these histone codes ranged from 1 to 112 nucleosomes with various patterns of multivalent histone modification signals. By the *K*-means clustering analysis, we found several major patterns of these codes (Fig. 3a) and representative examples of codes for genes in each cluster are shown in Additional file 1: Figure S5. Clearly, with single-nucleosome resolution of histone modification signals, we obtained much better clustering patterns than those low-resolution signals from traditional ChIP-Seq data, and these patterns were quantitative. We found that over 57% of genes were mainly regulated by three gene activation histone marks (c1 to c10 in Fig. 3b); meanwhile, we found that about 1/3 of genes were not regulated by these histone marks (c11 in Fig. 3b) and about 9% of genes were mainly regulated by H3K27me3 (c12/c13 in Fig. 3B). Two recent studies have shown that the low-resolution histone signals, the peak widths of H3K4me3, are associated with special groups of genes that are either tumor suppressors or are linked to cell identity and transcriptional consistency [21, 22]. Interestingly, with our high-resolution histone signals, we found that genes in each cluster mostly regulated different categories of functional pathways in the canonical pathways using Ingenuity (Fig. 3c), indicating that these epigenomic patterns could define specific functions in the biological processes in terms of epigenomic regulation.

### Functional and quantitative histone modification marks in regulating gene expression

**Fig. 4:**
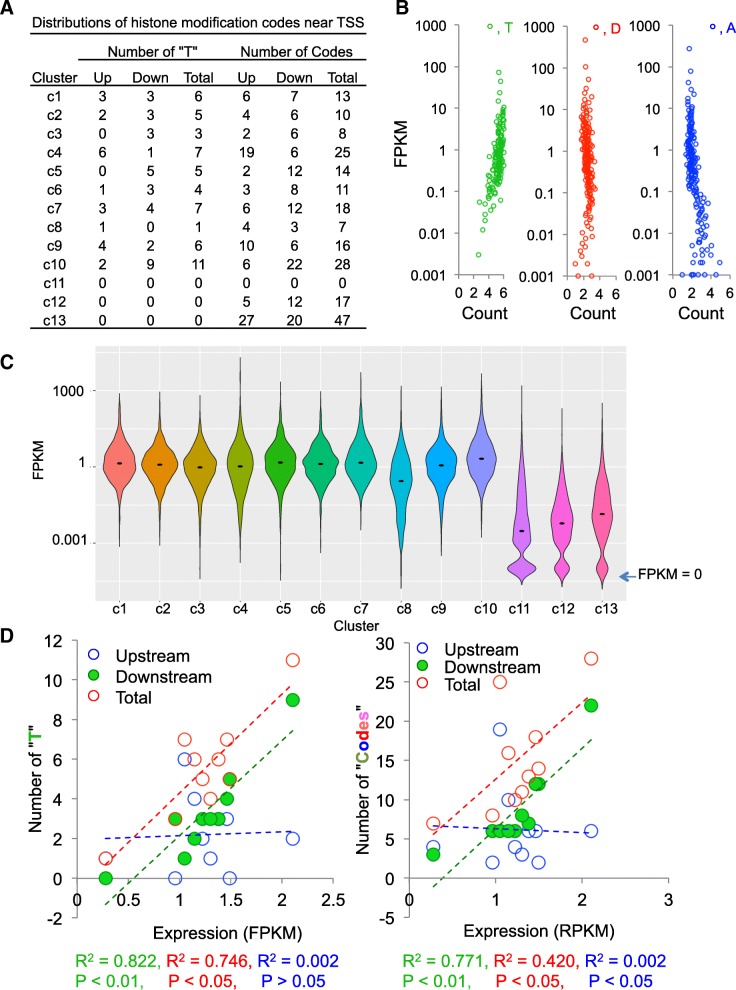
The correlation of histone modification codes with gene expression. (**a**) Distributions of the numbers of the trivalent histone mark “T” and all codes at the upstream (up), downstream (down), and combined both upstream and downstream (total) surrounding TSS. (**b**) Correlation of gene expression levels (RPKM) with the number of single and multivalent histone marks within ±2 kb of the TSS for all genes in HepG2 cells. T, H3K4me3/H3K9ac/H3K27ac; D, H3K4me3/H3K9ac; A, H3K4me3. (**c**) The violin plot of the expression values of all genes in each cluster. Black dots, the median expression levels. (**d**) The correlation between gene expression values and numbers of the trivalent histone mark “T” and all codes at the upstream, downstream, and combined both upstream and downstream (total) surrounding TSS. Gene expression values and histone counts are median values from Fig. 4c and a, respectively

Our single-nucleosome histone modification signals generated by NUCLIZE became quantitative histone modification data or allowed us to count the number of single or multivalent histone marks in each nucleosome. So, we counted the trivalent “T” and total histone codes at the upstream, downstream, or combined both upstream and downstream (total) of TSS for each cluster that we obtained in Fig. 3a; and Fig. 4a shows median values of these counts for all genes in each cluster. An obvious question is whether these quantitative histone marks have direct impact on gene expression. To address this, we first plotted the number of each multivalent histone mark, “T”, “D”, and “A” within ±2 kb of the TSS of each gene with its expression value (Fig. 4b); we found that those actively expressed genes had at least three or four “T”, regardless of low or high expression, indicating that a defined number of three or four trivalent marks may be required for activating gene expression as a threshold. However, “A” alone were highly accumulated in genes with low or no expression and did not have the similar regulatory patterns to those for “T” (Fig. 4b). These data indicate that a defined threshold (three or four) of “T” surrounding the TSS could be critical for determining gene activation.

Next, we plotted the gene expression values for all genes in each cluster (Fig. 3c) and found that genes in clusters from 1 to 10 (c1 to c10) had clearly active gene expression compared to those genes from clusters 11 to 13 (c11 to c13) having low or no expression. Interestingly, genes in cluster 8 (c8) showed lowest median expression levels and genes in cluster 10 (c10) showed the highest median expression levels, indicating that the more nucleosomes with active histone modification marks might lead to higher gene transcription. To further dissect this, we plotted the median gene expression values in each cluster with those histone counts in Fig. 4a. Although both gene expression values and numbers of “T” or “codes” in each correlation analysis showed statistical significance, the numbers of “T” in the downstream of TSS were mostly correlated to the gene expression levels in each cluster and the total numbers of “T” or all codes showed similar correlations with gene expression levels (Fig. 4d); however, neither numbers of “T” nor all codes in upstream of TSS showed any correlation with gene expression levels (Fig. 4d). These data suggest that at least three of “T” in the downstream of TSS could be essential for gene activation.

## Conclusions

Our study provides a novel toolkit for generating histone modification data at single-nucleosome resolution and makes them become quantitative epigenomics data using genuine nucleosome positions, which is critical for the future functional epigenomics studies. Two recently published approaches [23, 24] for directly mapping histone modification in isolated single nucleosomes from native chromatin are very promising for generating genuine quantitative epigenomics data, given the major defect in these approaches will be resolved soon, i.e., overnight incubation of native chromatin with antibodies during immunoprecipitation led to many false-negative signals; these approaches are fancy but difficult to be conducted in a regular laboratory for epigenomic studies at the current stage compared to our approach. Whether the epigenetic codes generated from our quantitative epigenomics data have specific functions in regulating the expression levels of genes or certain functional groups of genes would be worthwhile for future investigation. Regardless whether there are epigenetic codes or not, deeper analysis of the patterns of these quantitative epigenomics data would be helpful for revealing general principle for genuine functional epigenomic regulation, which, however, will require the analysis of large sets of quantitative epigenomic data using the NUCLIZE approach. Meanwhile, as most of other epigenomic studies, our findings are mainly correlations between potential epigenetic codes and gene expression but not causal relationship; nevertheless, with recent development of Cas9 fusion proteins of histone modification enzymes [25–28], full addressing the functions of these potential epigenetic codes is promising. More importantly, these quantitative epigenomics data make feasible for genuine comparison of epigenomic signals between two conditions, which would be essential for revealing the mechanisms underlying dynamic epigenomic regulation in biological processes. Thus, our NUCLIZE toolkit is a transitional but powerful approach for quantitative epigenomics studies in dealing with large amount of mapping data for histone modification using regular ChIP-Seq that deposited in the public databases and also practicable for current quantitative epigenomics studies.

## Availability and requirements

Project name: NUCLIZE.

Project home page: http://nuclize.mayo.edu

Operating system(s): Platform independent.

Programming language: C++.

Other requirements: Qt 5.4.0.

License: GNU GPL.

Any restrictions to use by non-academics: licence needed.

## Additional file

Additional file 1: Supplementary figures (**Figures** **S1 – S5**). Supplementary methods and materials. (PDF 3038 kb)

## Data Availability

All genomic data are deposited in GEO (GSE76344). The NUCLIZE algorithm can be accessed at https://nuclize.mayo.edu

## Abbreviations

ChIP-Seq: Chromatin immunoprecipitation coupled high-throughput sequencing
FDR: False discovery rate
MNase: Micrococcal nuclease
TSS: Transcription start site
